# Sustainability and Influencing Factors in Bacterial Cellulose Production: A Review of the Impact of Microorganisms, Culture Media and Cultivation Methods

**DOI:** 10.17113/ftb.63.03.25.8746

**Published:** 2025-09-05

**Authors:** Rebeca Priscila Flora Catarino, Vinicius Avanzi Barbosa Mascareli, Viviane Lopes Leite da Costa, Ana Clara Longhi Pavanello, Wilma Aparecida Spinosa

**Affiliations:** State University of Londrina, Celso Garcia Cid Road, Km 380, 86051-970 Londrina, PR, Brazil

**Keywords:** bacterial cellulose production, oxidative fermentation parameters, microbial selection, bioprocess, agro-industrial waste

## Abstract

This review provides a comprehensive analysis of bacterial cellulose production, with a focus on the key factors influencing the bioprocess, including microorganism selection, substrate optimization and cultivation techniques. It addresses the challenges associated with bacterial cellulose production and proposes strategies to optimize upstream processes, such as microorganism preservation, inoculum preparation and culture medium formulation, which are critical for improving production efficiency. Additionally, the review explores the effects of fermentation parameters such as time, temperature, and oxygen availability on the yield and quality of bacterial cellulose. It also highlights the growing interest in utilizing alternative substrates, particularly agro-industrial waste, to reduce production costs and improve sustainability. By evaluating these factors, this review provides valuable insights for the further development of bacterial cellulose production in both research and industrial applications.

## INTRODUCTION

The biotechnology industry produces numerous products from the metabolism of microorganisms that benefit humans, animals and the environment. Among these, bacterial cellulose has gained importance in industrial applications due to its versatility. Bacterial cellulose is a biodegradable, biocompatible biopolymer with high purity, and is classified as a Generally Recognized as Safe (GRAS) by the Food and Drug Administration (FDA) (*1*–*3*).

Since its discovery in 1886 during vinegar production, bacterial cellulose has consistently attracted global research interest, with efforts focused on increasing its production and improving its properties for high value-added applications (*4*, *5*). Potential applications of bacterial cellulose include food additives and dietary fiber-enriched products (*6*, *7*), food packaging materials (*8*) and bioengineering uses such as the entrapment of cells and bioactive compounds, the development of biosensors (*9*–*11*), cosmetics (*12*), wound dressings (*13*) and applications in electronics (*14*). Despite extensive research demonstrating its potential applications, the commercial use of bacterial cellulose remains limited due to high production costs. Advancing bacterial cellulose commercialization requires strategies to increase yield and reduce production costs, ultimately making this biopolymer economically viable for wider applications. To achieve this, optimizing the activity of microorganisms, nutrient sources, and fermentation techniques is critical to overcome these limitations.

From a metabolic point of view, bacterial cellulose is a product of oxidative fermentation by acetic acid bacteria. These microorganisms metabolize carbon sources, such as sugars, ethanol and sugar alcohols, to produce energy through a series of enzymatic reactions (*15*). Understanding the mechanisms and factors involved in bacterial cellulose formation is crucial for controlling fermentation parameters and optimizing metabolite production. In general, a fermentation process comprises three main steps: upstream steps (selection and preservation of microorganism, preparation of the inoculum, and formulation of the culture medium), the fermentation phase, and downstream steps (product purification and waste management). Each step directly or indirectly affects product yield, properties, bioprocess efficiency and cost-effectiveness.

Currently, researchers are focusing on isolating new strains with high bacterial cellulose productivity, optimizing culture media, and improving fermentation methods to enhance the efficiency and feasibility of bacterial cellulose production (*16*–*18*).

This review provides a comprehensive analysis of the key factors influencing bacterial cellulose production, focusing on upstream processes such as microorganism selection and culture medium optimization. Unlike previous studies that focus on isolated aspects of the bioprocess, this review integrates these factors into a detailed discussion of fermentation methods and their critical role in increasing production yield and cost-effectiveness. This review further highlights the importance of novel microbial strains and advanced fermentation techniques, and provides a broader perspective on strategies to improve the scalability of bacterial cellulose production and commercial viability. Additionally, it incorporates recent advances in the use of agro-industrial waste as substrates, reflecting current sustainability trends in biopolymer production.

## BACTERIAL CELLULOSE

Bacterial cellulose is an extracellular polysaccharide produced by microorganisms. Unlike plant cellulose, it is a highly pure form of cellulose with a higher degree of crystallinity, which makes it mechanically stronger and more suitable for specialized applications. While plant cellulose is a component of plant cell walls and typically contains impurities such as hemicelluloses and lignin, bacterial cellulose is synthesized in a controlled environment by microorganisms, ensuring superior purity and uniformity (*4*, *19*, *20*).

The first observation of bacterial cellulose dates back to 1886, when Adrian Brown reported the formation of a white, gelatinous film on the surface of the medium during acetic acid fermentation for vinegar production. At that time, bacterial cellulose was referred to as vinegar-plant or vinegar-mother. Later chemical and structural analyses confirmed its similarity to plant cellulose (*4*, *19*, *20*). Today, bacterial cellulose is also known as biocellulose, microbial cellulose, or bacterial nanocellulose.

The most important bacterial strains that produce cellulose belong to the acetic acid bacteria (AAB). *Komagataeibacter* genus, due to higher yields than other genera of AAB (*21*, *22*). In microorganisms, bacterial cellulose is a metabolite produced during acetic acid fermentation (oxidative fermentation) and supports flotation by acting as a cell support at the air-liquid interface. This mechanism, which is associated with aerobic metabolism, ensures cell survival under stress conditions. In addition, bacterial cellulose contributes to cell protection against dehydration, ultraviolet radiation and acetic acid diffusion to the cytoplasmic membrane (*23*–*26*). During vinegar production, large amounts of bacterial cellulose can become a problem as they require additional purification steps in the fermentors. Also, the presence of AAB in organic vinegar or remaining cells in conventional vinegar (not completely removed by filtration) can affect the visual appearance of the final product (*27*).

However, studies of the chemical composition and structural properties suggested that the biopolymer has the potential to produce biotechnological products after purification steps. Because of that, researchers have focused on understanding the mechanisms of bacterial cellulose synthesis, isolating bacterial cellulose-producing microorganisms, and optimizing production for controlled processes at both laboratory and industrial scales.

### Bacterial cellulose-producing microorganisms

Several microorganisms have been identified as producers of bacterial cellulose, such as *Aerobacter*, *Acetobacter*, *Komagataeibacter*, *Achromobacter*, *Agrobacterium*, *Alcaligenes*, *Azotobacter*, *Pseudomonas*, *Rhizobium* and *Sarcina* (*21*, *22*). Among the AAB species, *Komagataeibacter xylinus* is considered a model for the production of this biopolymer from different carbon and nitrogen sources due to the high bacterial cellulose yield. However, new species of *Komagataeibacter* have been continuously isolated for this purpose, including *Komagataeibacter medellinensis*, *Komagataeibacter intermedius*, *Komagataeibacter hansenii*, *Komagataeibacter europaeus* and *Komagataeibacter rhaeticus* (*28*–*31*). These microorganisms are usually isolated from kombucha, fruit, vegetables and vinegar (*32*–*36*). Apart from AAB, new genera and species of microorganisms have been reported as bacterial cellulose producers, for example *Bacillus licheniformis* (*37*), *Enterobacter* sp. FY-07 (*38*) and *Lactobacillus hilgardii* IITRKH159 (*39*).

Bacterial cellulose is mainly produced by Gram-negative bacteria, particularly by the genus *Komagataeibacter*, such as *Komagataeibacter xylinus*. These bacteria are efficient cellulose producers due to their specialized outer membrane and secretion systems, which facilitate the release of cellulose into the extracellular space. Strains like *Komagataeibacter rhaeticus* K3 and *Gluconacetobacter xylinus* have shown high cellulose yields, often utilizing simple sugars, such as glucose and sucrose. However, the production process requires optimized media and strict control of environmental conditions, such as pH and temperature, to achieve maximum efficiency. While Gram-negative bacteria tend to outperform Gram-positive species in bacterial cellulose productivity, the potential of Gram-positive bacteria has recently gained more attention (*40*).

As recently reported by Saleh *et al.* (*40*), Gram-positive bacteria, including *Lactiplantibacillus plantarum* AS.6, *Lactobacillus hilgardii* and *Bacillus velezensis*, also produced bacterial cellulose, although at a lower yield than Gram-negative strains. In the study, *Lactiplantibacillus plantarum* AS.6 was identified as a promising bacterial cellulose producer with a productivity rate of 56 %, which is higher than that of other Gram-positive species. When optimized, *L. plantarum* AS.6 can produce 4.51 g/L of bacterial cellulose, doubling the yield compared to the basal medium. This suggests that Gram-positive bacteria can be competitive in the production of bacterial cellulose when the growth medium is optimized. Additionally, *L. plantarum* AS.6 produced composites with strong antibacterial activity, indicating its potential for biomedical applications such as wound dressings and drug delivery.

### Acetic acid fermentation and bacterial cellulose synthesis

Bacterial cellulose is a product of oxidative metabolism. Acetic acid bacteria obtain energy through oxidative fermentation (acetic acid fermentation) pathway to compensate for the low energy yield of aerobic respiration and improve biomass formation through incomplete substrate oxidation (*41*, *42*). During acetic acid fermentation, organic substrates such as ethanol, glucose, organic acids, and polyols, are incompletely oxidized to CO_2_ and H_2_O. The residual products of this metabolism are used in the biotechnology industry to produce high value-added products (*e.g.* ketones, organic acids and exopolysaccharides, such as bacterial cellulose) (*43*).

Bacterial cellulose biosynthesis is a highly precise and specific process controlled by catalytic and regulatory enzymatic complexes using uridine diphosphate glucose (UDP-glucose) as a precursor (*44*). In this mechanism, the first step is the phosphorylation of glucose to glucose-6-phosphate by the enzyme glucokinase. Then, the phosphoglucomutase promotes the isomerization of glucose-6-phosphate to glucose-1-phosphate, which is converted to UDP-glucose by uridine diphosphate pyrophosphorylase. The polymerization of glucose from UDP-glucose to β-glucan chains is catalyzed by cellulase synthase, a complex of four subunits, namely the bacterial cellulose synthase subunits A, B, C and D (*BcsA*, *BcsB*, *BcsC* and *BcsD*), coded by three (*bcsAB, bcsC* and *bcsD*) or four (*bcsA*, *bcsB*, *bcsC* and *bcsD*) genes. Finally, the β-glucan chains are crystallized into cellulose (*22*, *45*, *46*).

**Fig. 1:**
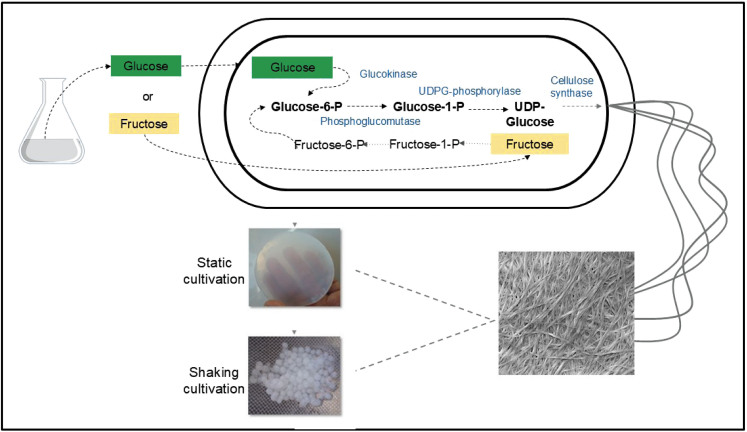
Biosynthesis of bacterial cellulose from glucose and fructose. UDP=uridine diphosphate glucose, P=phosphate

The synthesized chains are secreted into the medium through pores in the cell of the microorganism, which enables the elongation and association of the chains in the extracellular medium and leads to bacterial cellulose subfibrils (1.5 nm wide). These subfibrils give rise to nanofibrils (3–4 nm thick), which eventually form cellulose ribbons (40–60 nm wide and 3–8 nm thick). The random arrangement of the bacterial cellulose ribbons leads to a three-dimensional, porous and highly crystalline network (*3*, *4*, *21*, *47*, *48*). The material observed in the culture medium can have different shapes depending on the cultivation method, the strain and nutrient sources (Fig. 1).

### Structure, function and application of bacterial cellulose

As previously mentioned, bacterial cellulose has a similar structure to plant cellulose. Cellulose consists of β-glucopyranosyl units linked by β-(1→4) glycosidic bonds that form a long-chain polymer with a degree of polymerization greater than 20 000 (*49*). The association between the β-glucopyranosyl units results in a planar structure that forms a ribbon. This planar and linear structure allows the formation of fibrous and polycrystalline bundles along extensive zones due to the association between cellulose molecules by hydrogen bonds. These structures contain both amorphous and crystalline zones (*49*–*51*).

In recent years, the production of cellulose from microorganisms has been the subject of research in several countries since the fermentation process produces a material with superior properties and high purity, allowing its application in products such as food, biomedicine and pharmaceuticals (*16*, *52*, *53*). The structure of bacterial cellulose formed by the three-dimensional network provides remarkable properties, namely high mechanical strength, crystallinity, stability to chemical agents and high temperatures, high water retention capacity and resistance to degradation. Bacterial cellulose is free of lignin and hemicellulose, it does not require intense purification, and is also a biocompatible and biodegradable biopolymer (*21*, *54*, *55*).

High crystallinity is one of the main characteristics of bacterial cellulose, with the degree of crystallinity ranging from approx. 60 to 90 %, depending on the cultivation conditions and the ability of the strain to convert the substrate, as well as the adaptation to the fermentation system. Crystallinity influences other characteristics of the biopolymer such as mechanical properties and thermal stability (*34*, *56*, *57*). Concerning the crystalline structure, cellulose I (Iα and Iβ) and cellulose II forms are frequently obtained in the fermentation culture. The Iα (triclinic) and Iβ (monoclinic) forms correspond to crystalline structures and differ in the distribution of intra- and interunit hydrogen bonds. In cellulose II, the random arrangement of the chains results in highly amorphous regions, which also differ in their high thermodynamic stability. In most cases, a higher crystallinity is observed in static culture, while amorphous content is more pronounced in agitated cultivation (*21*, *58*, *59*).

The large surface area, the high number of hydroxyl groups, and its porosity enable bacterial cellulose to interact with water and polymers, allowing the application as a support material for the immobilization of enzymes, cells and nanoparticles. Bacterial cellulose has a high water-holding capacity (WHC) and can retain approx. 90 % of its mass. This property is due to the strength of the hydrogen bonds involved in the adsorption of water molecules on the surface of the fibers and the density of the bond between the crosslinked fibers. The presence of thin and long ribbons in the structure of bacterial cellulose also explains its greater water retention capacity, moldability and high tensile strength (*30*, *60*–*62*). Regarding these properties, bacterial cellulose has potential for food, bioengineering, cosmetics, biomedical and electronic fields (Table 1 (*7*, *19*, *46*, *63*–*82*)).

**Table 1:** Properties of bacterial cellulose and potential application fields

Application	Function	Structure/property	Reference
Meat products, ice cream	Fat replacer	Water-holding capacity, emulsion stabilization, amphiphilic nature	(*63*–*65*)
Dietary fiber source, low calorie products, low cholesterol diet	Functional food ingredients	Insoluble dietary fiber, high water-holding capacity, ion exchange capacities	(*7*, *19*, *46*)
Food packaging, edible films and coatings, active and intelligent packaging film; immobilization of cells, enzymes and antimicrobial agents	Food packaging and support for bioactive compounds	High surface area, porosity, high pore volume, gelling behavior, high crystallinity, hydrophilicity, rehydration property; chemical, thermal and mechanical stability; barrier properties	(*19*, *66*–*68*)
Pickering emulsion, edible foam, beverages, bakery products, dairy products	Thickener and stabilizing agent in emulsion, suspensions, and foam stabilizer	Amphiphilic nature, high surface area, crystallinity, three-dimensional structure	(*69*–*75*)
Bioengineering, tissue engineering	Controlled release systems, biosensors, scaffold for regeneration, vessel substitute	High purity and crystallinity, porosity, biocompatibility, nanofibrillar matrix, mechanical strength, durability, flexibility, elasticity	(*76*–*78*)
Cosmetics	Bioactive compound delivery	High water-holding capacity, biocompatibility, nanofibrillar porous structure	(*79*)
Wound dressings, drug delivery systems	Controlled release systems, water retainer	High water absorption capacity, biocompatibility, porosity, crystallinity, thermal stability	(*80*, *81*)
Electronic field	Flexible substrates for electronic devices, conductive materials and biosensors	High purity and crystallinity, porosity, biocompatibility, nanofibrillar matrix, mechanical strength, durability, flexibility, large surface area	(*78*, *82*)

The material can be used in different shapes, for example, as nanofibers, nanocrystals, dried or wet pellicles and spheres. In food applications, bacterial cellulose acts as a multifunctional ingredient and its addition to food products does not affect the sensory properties since it can be coloured and flavoured (*69*, *83*). From a nutritional point of view, cellulose has a health-promoting function and can be used as a source of dietary fiber and for low-calorie or gluten-free products. As food additives, these biopolymers have been suggested as stabilizers, thickeners and texture modifiers (*1*, *3*).

The three-dimensional, porous and crystalline structure as well as the presence of hydroxyl groups in the surface area allow the association of bacterial cellulose with other polysaccharides and proteins through hydrogen, van der Waals and hydrophilic bonds, improving their functionalities. Otherwise, the crystalline structure facilitates hydrophobic interactions, ensuring the amphiphilic properties of bacterial cellulose (*63*, *70*). The formation of nanocomposites is useful for the development of biodegradable food packaging and edible films. In this case, bacterial cellulose could be combined with other polymers, proteins, bioactive composites and inorganic nanoparticles to improve mechanical and thermal properties, barrier performance, and antimicrobial properties (*84*–*86*). Some research papers report the use of chemical, mechanical and enzymatic modifications to improve or develop specific properties that enable further applications (*63*–*65*, *87*). In addition to various fields of application, the low yield and high production costs are a limiting factor. In this context, it is crucial to understand and optimize the production parameters to improve the cost-effective bioprocess.

**Fig. 2:**
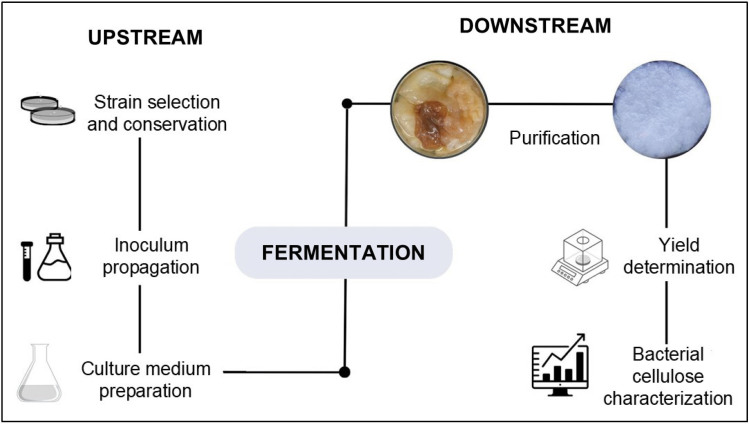
Scheme of bacterial cellulose production

The production of bacterial cellulose comprises three stages: *i*) upstream, *ii*) fermentation and *iii*) downstream. Upstream production includes strain selection and conservation, preparation of the inoculum and medium, and determination of cultivation conditions. During fermentation, several parameters can affect the yield and product properties, for example pH, oxygen, temperature and agitation, so they should be controlled. On the other hand, the downstream steps require product purification, neutralization, characterization and effluent treatment. A summarized scheme of bacterial cellulose production is shown in Fig. 2.

Different strategies can be used to overcome or minimize limiting factors in each step of the process to optimize bacterial cellulose production. The following sections discuss the characteristics of the key factors of bacterial cellulose production (microorganism, substrate and cultivation methods), including some strategies to improve this bioprocess.

## ACETIC ACID BACTERIA FOR BACTERIAL CELLULOSE PRODUCTION

The cultivation of bacterial cellulose producers is a crucial step when considering the nutritional requirements for cell metabolism and the adaptation of microorganisms to fermentation conditions. In this context, researchers are constantly looking for new strains with a high capacity to produce bacterial cellulose. AAB strains are fastidious microorganisms to cultivate and isolate, posing challenges for researchers to find alternatives for cellulose production (*88*–*90*). *Komagataeibacter* species have previously been described as the most efficient cellulose producers as they are able to grow and produce cellulose from different carbon and nitrogen sources (*91*, *92*). *K. intermediuns, K. rhaeticus, K. hansenii* and *K. medellinensis* are often referred to as high producers of bacterial cellulose, similar to the strain *K. xylinus*, the model for bacterial cellulose production. The main sources for the production of bacterial cellulose are vinegar (*33*, *93*), kombucha (*94*), fermented juices (*29*) and fruit and vegetables (*15*).

The producers of bacterial cellulose have developed mechanisms to survive under stressful conditions, usually in the isolation environment, for example, resistance to high acid and ethanol concentrations. All these mechanisms make AAB attractive for industrial processes and are useful to improve the formation of biopolymers and increase the rate of bioprocess design (*92*, *95*). Moreover, some *Komagataeibacter* strains showed a remarkable ability to produce cellulose under alkaline conditions, which could be related to the protective nature of the material (*29*, *96*). Additionally, resistance mechanisms have been investigated under different cultivation modes, such as static and agitated cultivation, and the adaptation to the highest rotational speed is referred to as a strain-dependent property (*91*). Cultivation under laboratory conditions and in synthetic media can reduce the resistance of microorganisms, suggesting that these mechanisms are inducible or transient (*97*, *98*). However, product formation must be evaluated considering the interaction between strain, culture medium and cultivation method once the performance of the microorganisms is affected by the adaptation of the cells to the cultivation conditions. In this case, the same strain cultivated in different bioprocesses does not always give the same yield and productivity (Table 2 (*17*, *29*, *30*, *56*, *93*, *99*–*102*)).

**Table 2:** Acetic acid bacteria performance on bacterial cellulose (BC) production under different conditions

Strain	Source	Nutrient source	Method	Parameter		*γ*(BC)/ (g/L)	*Q*(BC)/ (g/(Lh))	Reference
				*t*/°C	Time/h			
*K. europaeus* SGP37	Rotten grapes	Hestrin-Schramm (glucose)	Static	30	384	5.61	0.0146	(*101*)
		Hestrin-Schramm (fructose and ethanol)	Static	30	384	9.98	0.0260	
		Sweet lime pulp waste	Static	30	384	6.30	0.0164	(*102*)
		Sweet lime pulp waste supplemented with HS	Static batch	30	384	26.20	0.0682	
		Sweet lime pulp waste supplemented with HS	Static intermittent fed-batch	30	384	38.00	0.0990	
*K. intermedius* BCRC 910677	Fermented fruit juice	Hestrin-Schramm	Static	28	120	1.20	0.0100	(*29*)
		Synthetic optimized medium	Static	28	144	3.91	0.0271	(*100*)
*K. intermedius* V-05	Vinegar	Soy molasses with ethanol	Static	30	336	10	0.0297	(*93*)
		Hestrin-Schramm	Static	30	336	3.7	0.0110	
		Synthetic with amino acids (optimized)	Static	30	240	3.02	0.0125	(*17*)
*K. rhaeticus* AF-1	Kombucha tea	Hestrin-Schramm with ethanol	Static	28	96	6.70	0.0698	(*56*)
		Cashew tree exudate	Static	28	168	2.80	0.0167	(*99*)
		Cashew gum	Static	28	168	2.30	0.0137	
		Hestrin-Schramm	Static	28	168	~6.0	0.0357	
		HSCTE	Static	28	168	~6.0	0.0357	
		HSCG	Static	28	168	~6.0	0.0357	
		Sugarcane molasses- supplemented	Static	30	120	3.46-4.01	0.0288-0.0334	(*30*)
		Sugarcane molasses	Static	30	120	1.90	0.0158	
		Hestrin-Schramm	Static	30	120	3.00	0.0250	

HSCTE=Hestrin-Schramm cashew tree exudate medium, HSCG=Hestrin-Schramm cashew gum medium

Stress conditions can adversely affect cell metabolism and bacterial cellulose production. These factors can induce spontaneous mutations in the cells, leading to atypical cell morphology and growth, and they can also inactivate essential enzymes for polymer synthesis, thus reducing yield and material properties (*24*, *102*). In addition to reducing bacterial cellulose production, the effect of cells that cannot produce cellulose on the polymer structure can be investigated, with crystallinity being the most affected (*103*). Additionally, the presence of mutant cells affects the fiber assembly due to the formation of soluble polysaccharides, such as acetan, as both use the same starter molecule, UDP-glucose (*104*). Regarding the activity of the microorganisms, it is also important to consider the effects of the cultivation parameters on the formation of mutant cells. Changes in process parameters, pH, temperature, culture medium volume and oxygen availability affect cell metabolism and product formation (*105*).

Similarly, stress conditions, such as high sugar concentration, anaerobic conditions and high temperatures, can induce the viable but non-culturable (VBNC) state, which affects cell monitoring and product formation. Briefly, in the VBNC state, cells are alive, but do not grow on conventional media (nonselective) used to form colonies. The main metabolic and morphological characteristics affected are the modification of cell wall components, reduction in respiration rate, nutrient transport and macromolecular synthesis. Nevertheless, cells in the VBNC state are more resistant to physicochemical stress and antibiotics. A change in the environmental conditions and the composition of the medium could favor the cell growth and reverse this cell state (*41*, *106*, *107*).

To improve material formation, cellulose-producing bacterial strains can also be obtained using genetic engineering methods. The most important strategies include the modification of the *acs* operon, responsible for cellulose synthesis, and genes like *pgi* and *zwf*, which improve carbon metabolism to produce bacterial cellulose precursors. Disruption of competing pathways, such as polyhydroxybutyrate (PHB) synthesis, and regulation of cyclic dimeric guanosine monophosphate (di-GMP) amounts have also significantly improved production efficiency. These modifications use advanced synthetic biology techniques to optimize bacterial strains for industrial applications (*108*–*110*). Yang *et al*. (*111*) developed a recombinant strain for bacterial cellulose production in mannose-rich media by introducing genes from the *Escherichia coli* K-12 strain, which increased bacterial cellulose production by 84 % compared to the wild-type strain. Jacek *et al*. (*112*) modified the motility and cell size of *K. hansenii*, which are believed to influence the yield and network organization in the bacterial cellulose structure. The use of genetic engineering resulted in thicker ribbons of cellulose arranged in looser networks, and the biopolymer is proposed for the production of scaffolds.

### Monitoring and control of cell growth

The isolation, cultivation and cell preservation are crucial steps in the bioprocess due to their influence on the formation of biopolymers. The success of AAB cultivation for bacterial cellulose production has been associated with the nutritional requirements of the microorganism and the control of cell growth (*27*, *113*).

Culture media for microorganisms are classified based on their composition, such as chemically defined (synthetic), complex, selective, differential, and enrichment media. The use of complex media (composition not precisely known) is a strategy to evaluate the growth characteristics of unknown strains or to create an environment that can meet the complex nutritional requirements of some microorganisms (*114*).

Culture media for AAB isolation, pre-activation and inoculum propagation are formulated to satisfy nutritional demands and contain components that simulate the properties of the isolation environment, such as high sugar content, acetic acid and ethanol, which are found in fermentation bioreactors, fruit, vinegar or fermented beverages—common sources for AAB isolation. The main elements for cell formation are carbon, nitrogen, hydrogen, oxygen, sulfur, and phosphorus since these components are used for the synthesis of proteins, nucleic acids, carbohydrates and lipids (*114*, *115*).

Carbon and nitrogen are the most important nutrients due to their structural role in various cellular components (*17*). Carbon is an essential element for the synthesis of organic components in cell metabolism and energy production. In the culture medium, this macronutrient is provided by sugars, which constitute a large percentage in the formulation. Although AAB can metabolize several carbon sources, ethanol, glucose, mannitol and glycerol are the most common substrates for these metabolic pathways since they are oxidized by the membrane-bound (periplasmic) dehydrogenases and do not require previous hydrolysis reaction, which would imply additional cell work. The most important dehydrogenases in the oxidative fermentation of carbon sources include, for example, pyrroloquinoline quinone-dependent alcohol dehydrogenase (PQQ-ADH) and aldehyde dehydrogenase (ALDH), which oxidize ethanol to acetaldehyde and further to acetic acid, respectively. Additionally, PQQ-dependent glucose dehydrogenase (PQQ-GDH) oxidizes glucose to d-glucono-δ-lactone, while PQQ-glycerol dehydrogenase (GLDH) oxidizes polyols to ketones (*41*, *42*, *116*).

Nitrogen is required in the medium for the production of proteins, enzymes, nucleic acids and formation of biomass. For the cultivation of AAB and production of bacterial cellulose, this nutrient is supplied by organic (yeast extract, peptone, malt extract and amino acids) and inorganic sources, for example, ammonium sulfate and ammonium nitrate (*17*, *100*, *117*).

Minerals and vitamins also play essential roles in cell growth. Mineral components affect enzyme activity, nitrogen fixation and electron transfer from substrate to oxygen. Some essential minerals for AAB metabolism are molybdenum, boron and manganese. In addition, vitamins such as *p*-aminobenzoic acid, pyridoxine (B_6_), cyanocobalamin (B_12_), nicotinamide (B_3_) and ascorbic acid have shown significant effect on cell growth and bacterial cell production (*115*, *118*, *119*).

Table 3 (*18*, *33*, *97*, *116*, *120*–*124*) shows the main culture media most commonly used for the cultivation of AAB and their composition. These culture media are mainly composed of sugar (carbon source) and yeast extract or peptone (nitrogen source). Additives are often added to the culture media to supply the microorganism with nutrients. For example, ethanol has been proposed as an alternative energy source for microorganism growth, which also supports cell recovery from the viable but non culturable (VBNC) state and inhibits the non-producing cells (*34*, *125*). Similarly, organic acids such as acetic, citric, malic, lactic, pyruvic and succinic acids could be metabolized by AAB and used as intermediate metabolites for energy production (*116*, *126*).

**Table 3:** The main culture media for the isolation, cultivation and preservation of acetic acid bacteria

Culture medium	(*m*(compound)/*V*(medium))/%	Function	Reference
Acetic acid-ethanol (AE)	Glucose 1.5, yeast extract 0.2, peptone 0.3, ethanol 2.0, acetic acid 6.5	Isolation and enrichment	(*120*)
Glucose-yeast extract- carbonate (GYC)	Glucose 10, yeast extract 1.0, CaCO_3_ 2.0	Isolation and enrichment	(*97*)
Hestrin-Schramm (HS)	Glucose 2.0, yeast extract 0.5, peptone 0.5, Na_2_HPO_4_ 0.27, citric acid 0.115	Isolation, cultivation and bacterial cellulose production	(*18*, *33*, *116*, *121*)
Mannitol, yeast extract, peptone (MYP)	Mannitol 2.5, yeast extract 0.5, peptone 0.3	Isolation and enrichment Preservation	(*116*)
Carr	Yeast extract 2.5, ethanol 2.0, peptone 0.02	Preservation	(*122*)
Glycerol	Glycerol 15	Preservation	(*123*)
Malt extract	Malt extract 20	Preservation	(*124*)

In addition to medium composition, alternative methods of preparation of solid media, such as double-layer agar, can be used during isolation or enrichment stages. For AAB strains from industrial vinegar production, this method simulates the growth conditions in fermentation tanks (*116*). This technique was described by Entani *et al*. (*120*) and consists of creating an bottom layer with broth containing 0.5 % agar. The surface is then coated with the broth containing 1.0 % agar. The use of double-layer agar plate creates an environment with a high humidity that favors the growth of acidophilic colonies (*116*, *120*).

#### Alternatives to cell enumeration

Estimating the cell population is essential for understanding the growth profile of microorganisms, metabolic aspects related to bacterial cellulose production and fermentation control. The cell growth profile typically consists of four phases: lag, exponential (log), stationary, and death. In the lag phase, the cell count increases slowly as the microorganism adapts to the cultivation conditions. The exponential growth phase reflects the most intense cell activity and substrate consumption. During the stationary phase, nutrient availability is reduced and the growth rate equals the death rate. However, the cells remain active and continue to produce metabolites, such as bacterial cellulose. Eventually, the nutrient limitation leads to cell death (*127*, *128*). Traditionally, the AAB population is determined by cell enumeration in plating or by microscopy, as well as by turbidimetric and gravimetric methods. Since these techniques are well established in the production of vinegar and fermented beverages, they could also be used in the production of bacterial cellulose to improve the control of cell growth and product formation. However, alternative methods are useful for monitoring cell growth and analyzing the behavior of the microorganisms. These techniques should be applicable in fermentation routine to monitor cell growth and ensure the control of bioprocess.

Plating methods for cultivation and enumeration of AAB in synthetic culture media could be affected by the presence of cells in the VBNC, which leads to an underestimation of cell count and limits cultivation, isolation and cell maintenance (*116*, *129*, *130*). The VBNC state has been associated with discrepancies between the target inoculation rate and plate count results in fermentation systems. This state can interfere with the direct correlation between biomass formation, substrate consumption, and product yield, as VBNC cells cannot be enumerated (*41*, *130*–*132*).

Another limiting factor in cell determination is the attachment of the cells to the bacterial cellulose during inoculum propagation and fermentation. During preparation of the inoculum on a liquid medium, simultaneous cell growth and bacterial cellulose formation result in the cells remaining within the biopolymer structure. An alternative to overcome this limitation is the use of cellulase to release the cells attached to bacterial cellulose fibers, thereby increasing the number of free cells in the liquid medium and improving cell enumeration (*113*, *133*).

In addition to monitoring growth, it is crucial to use rapid methods to quantify and identify cell state (*i.e.* live, dead, or VBNC). Several techniques have been used to quantify both live and dead cells in acetic acid fermentation. Fluorescence has proven useful for this purpose, and shows good results for AAB enumeration compared to the plating method (*113*). Similarly, flow cytometry has been used to assist in cell enumeration during acetic acid fermentation (*134*) and to assess cell viability after exposure to stress factors (*125*). Additionally, real-time polymerase chain reaction (RT-PCR) offers an alternative to traditional AAB enumeration methods (*12*, *135*).

## DESIGNING CULTURE MEDIA FOR BACTERIAL CELLULOSE PRODUCTION

Culture media have a significant impact on the total cost of bacterial cellulose production and require strategies to overcome this limitation and increase the economic feasibility of the bioprocess (*126*, *127*). The conventional medium used for bacterial cellulose production was developed by Hestrin and Schramm (*121*). The composition of Hestrin-Schramm (HS) medium consists of (%, *m*/*V*): glucose 2, peptone 0.5, yeast extract 0.5, anhydrous sodium phosphate 0.27 and citric acid 0.115. The composition of the culture medium must provide sufficient macro- and micronutrients for cell growth and biopolymer synthesis. Considering the HS composition, each component plays an essential role in the metabolism of the microorganisms and bacterial cellulose formation. Carbon is supplied by glucose, which is the ideal precursor for the formation of bacterial cellulose chains (*4*, *12*, *136*). Peptone and yeast extract provide amino acids for protein synthesis and essential compounds, such as vitamins and minerals, for the growth of microorganisms (*117*, *137*, *138*). Finally, anhydrous sodium phosphate and citric acid exert a buffering effect during cell cultivation (*139*).

Increasing bacterial cellulose yield and reducing medium costs are essential for bioprocess viability. Nowadays, different approaches have been used to develop economically feasible nutrient sources for bacterial cellulose production. These strategies include the modification of individual components of standard media, the supplementation of culture media, the formulation of synthetic media and the use of low-cost materials. All approaches must consider an ideal carbon and nitrogen ratio for bacterial cellulose production. In microbial biopolymer production, excess nitrogen increases biomass formation while limiting biopolymer production, while an excess of carbon over nitrogen decreases protein synthesis and reduces microorganism growth. Thus, the energy from the excess carbon is used to produce the polysaccharide (*28*, *140*, *141*).

Many studies on bacterial cellulose have reported higher production when the carbon and nitrogen sources in the HS medium were modified by changing the concentration or type of sources. Basu *et al.* (*142*) used response surface methodology to determine the optimal HS composition for the *G. hansenii* strain. In this case, glucose and sucrose were analyzed at different concentrations and the authors found higher bacterial cellulose yields with sucrose, a cheaper carbon source than glucose. Similarly, Jacek *et al*. (*94*) reported an increase in bacterial cellulose production when glucose was replaced by eucalyptus biomass hydrolysate in the HS medium supplemented with ethanol. Considering the effect of nitrogen on cell growth and biopolymer formation, Santoso *et al*. (*100*) used different nitrogen sources, namely, yeast extract, peptone, malt extract and ammonium sulfate in HS medium. The results suggested that peptone was the most suitable source for *K*. *intermedius* (BCRC 910677), while no bacterial cellulose was produced when ammonium sulfate was used as N substitute.

The formulation of synthetic media is another alternative to improve the bacterial cellulose yield from different strains. Gomes *et al.* (*17*) evaluated the effect of amino acid supplementation on the metabolism of *K. intermedius* V-05 for bacterial cellulose production. The authors reported aspartic acid (1.5 g/L), phenylalanine (1.5 g/L) and serine (3.0 g/L) as essential elements in the formulated medium (50 g/L sucrose, 10 g/L (NH_4_)_2_SO_4_), 2 g/L Na_2_HPO_4_, 1 g/L MgSO_4_7H_2_O and 10 mL/L ethanol) and obtained 3.02 g/L from the optimized medium.

In recent years, the use of low-cost materials, especially agro-industrial waste, for the biosynthesis of bacterial cellulose has attracted much attention. These materials not only reduce production costs, but also contribute to environmental sustainability by utilizing waste that would otherwise be discarded. Several agro-industrial by-products have been successfully used as substrates for bacterial cellulose production, *e.g.* cashew apple juice, soybean molasses (*119*), potato peel waste (*143*), sugar beet molasses, cheese whey, tobacco waste (*20*), oat hulls (*144*) and brewing by-products (*e.g.* beer and distillery waste) (*145*).

These waste materials provide a rich source of carbon and nutrients necessary for bacterial growth and cellulose synthesis. However, their complex and variable compositions pose a challenge to the fermentation process. The undefined nature of these substrates can lead to inconsistencies in the bioprocess, making it difficult to achieve reproducible results and potentially affecting the quality and yield of the bacterial cellulose produced. For example, the presence of inhibitors or non-fermentable components in these waste materials can hinder bacterial growth or cellulose production efficiency.

To overcome these challenges, some agro-industrial waste materials require pre-treatment processes such as acid or enzymatic hydrolysis to break down complex polysaccharides and increase the concentration of fermentable sugars. While these treatments can improve bacterial cellulose production, they also incur additional costs and can complicate the overall process. Furthermore, extensive purification may be necessary to remove residual contaminants, which further adds to the operational cost (*114*, *144*).

The development of an optimal culture medium for bacterial cellulose production requires careful consideration of cost-efficiency, environmental sustainability and desired application outcomes. Agro-industrial residues, such as cantaloupe peels (*146*), starchy kitchen waste (*147*) and paper sludge (*148*), have shown great potential as alternative substrates for the biosynthesis of bacterial cellulose. The enzymatic hydrolysis of these substrates increases the availability of fermentable sugars, which significantly increases bacterial cellulose production. For instance, hydrolyzed cantaloupe peels produced a bacterial cellulose yield of 3.49 g/L, while starchy kitchen waste hydrolysates yielded 2.11 g/L. Similarly, paper sludge enzymatically processed in a fed-batch system increased bacterial cellulose production to 3.10 g/L, outperforming batch fermentation. These results highlight the feasibility of using waste-derived substrates to reduce production costs and minimize the impact on the environment, while offering pathways to valorize cellulosic and starchy wastes (*146*–*148*).

Optimization of medium formulations using techniques such as Box-Behnken design (BBD) can further improve bacterial cellulose production and application-specific performance. For instance, BBD optimization of the hydrolysis of starchy kitchen waste has maximized the availability of reducing sugars, allowing researchers to adapt culture media to achieve sustainable bacterial cellulose production with improved functionality for various applications, including wastewater treatment, biomedical materials and environmental remediation (*146*–*148*).

On the other hand, the use of defined (synthetic) media offers advantages in terms of process control, reproducibility and scalability. These media have a known and consistent composition, which enables better monitoring and optimization of fermentation parameters. Additionally, the use of defined media can simplify the recovery and purification steps, resulting in higher quality bacterial cellulose production. However, synthetic media are generally more expensive than agro-industrial waste, which can offset some cost-reduction benefits (*114*, *144*).

Despite the limitations, the exploration of agro-industrial waste as an alternative culture medium for bacterial cellulose production remains a promising area of research. With further optimization and pretreatment strategies, agro-industrial by-products can serve as a sustainable and cost-effective source for bacterial cellulose production, thus contributing positively to economic and environmental goals. Additionally, bacterial cellulose produced from waste materials could have higher added value in various applications, such as biocomposites, packaging and medical products, making it a potential key player in the circular economy (*20*, *147*, *149*).

## CULTIVATION METHOD

Bacterial cellulose can be fermented under static or agitated cultivation, and the method used influences both the yield and the material properties. However, the success of each method depends on the adaptation of the strain and the interaction of these variables with the culture medium. Under static conditions, the AAB are inoculated in fermentation flasks or bioreactors with sterile culture medium and incubated at predefined temperature and time conditions. In this method, the bacterial cellulose forms at the air-liquid interface as a gelatinous pellicle, shaped according to the flask used for cultivation (*150*).

Although static cultivation is the most commonly used technique for bacterial cellulose production, agitated (stirred or shake) culture has been proposed as an alternative as it can overcome some limitations of the static method. In agitated cultivation, the culture medium inoculated with AAB is incubated under different agitation speeds, and the biopolymer is synthesized as ellipsoidal, stellate, or fibrous components dispersed in the culture medium (*1*). Compared to static cultivation, crystallinity is the most important characteristic affected by agitation, particularly at high rotation speeds. This parameter reflects the structural organization, which would be affected by the shear force, resulting in a less organized network. The formation of spherical bacterial cellulose under the agitation system results from cell aggregation around air bubbles, following a ribbon-like arrangement. However, the mechanism is also influenced by the inoculum, the carbon sources and the temperature of the medium volume (*59*, *151*).

Saleh *et al.* (*152*) reported in their work that bacterial cellulose production is highly influenced by fermentation conditions, with static fermentation consistently outperforming agitated fermentation in terms of yield. The addition of hydroxyapatite nanoparticles to the culture medium further increased bacterial cellulose production, with static conditions yielding 4.10 g/L, approx. 1.25 times higher than agitated fermentation. Static fermentation supports BC formation at the air-liquid interface, optimizing oxygen availability, while agitation can cause excessive oxygen diffusion, genetic instability and lower yields. Additionally, structural analysis of bacterial cellulose/hydroxyapatite composites confirmed improved functional properties, especially under static conditions, highlighting their potential for biomedical applications like bone tissue engineering due to improved cell viability and attachment (*152*).

The evaluation of the strain performance under both systems should consider the effect of the culture medium on cell viability and the ability of cells to adapt metabolic mechanics under each cultivation method used. Therefore, it is useful to consider the medium composition and strain when comparing the static and agitated methods. The main differences observed in studies comparing both methods, where the same strain and nutrient sources were considered in both cultivations, include yield, crystallinity index, water retention, porosity, and bacterial cellulose form (Table 4 (*24*, *34*, *71*, *103*, *153*)).

**Table 4:** Bacterial cellulose production by acetic acid strains under static and agitated cultivation

Strain	*γ*(bacterial cellulose)/(g/L)		Bacterial cellulose properties		Reference
	Static	Agitated	Static	Agitated	
*A. xylinum* BCA263 *K. xylinus BCC529* *G. xylinus* P1	3.97 2.48 1.40	1.70 1.66 1.72	Higher crystallinity, stronger tensile strength, denser network structure, higher temperature resistance	Larger pore sizes, lower crystallinity, higher water retention	(*71*)
*K. xylinus* (KX) *K. xylinus* (TISTR 086) *K. xylinus* (428) *K. xylinus* (975) *K. xylinus* (1011)	1.14–1.84 0.14–0.39 0.09–0.22 1.11–1.55 0.57–1.46	0.60-1.20 (~) 0.00-0.10 (~) 0.20-0.40 (~) (~) 2.40-3.54 (~) 3.20-4.69	Higher crystallinity and smaller crystallite sizes	Disorderly reticulated structures of microfibrils, higher cellulose Iα content in the flocky asterisk-like bacterial cellulose than in the solid sphere-like cellulose	(*103*)
*Komagataeibacter* sp. nov. CGMCC 17276	8.85	3.22	Higher crystallinity, high water-holding capacity, denser network	Network structure looser and more porous, higher porosity	(*153*)
*K. hansenii* JR-02	4.62	3.14	Thicker fibers, higher thermal degradation temperature and lower moisture content, higher crystallinity	Higher mass loss, higher moisture content and amorphous proportion	(*34*)
*G. hansenii* P2A	1.89	3.25	Ordered and dense network of fibrils with (8–10 nm diameter), the network was composed of interconnected layers	Slight decrease in the crystallinity index, looser clump of disordered short and thin fibrils, lower molecular mass, increased thermal stability due the gradual increase in Iβ phase content	(*24*)

These aspects are essential to define the final biopolymer application (*16*, *71*). Bacterial cellulose production is influenced by surface area, volume of culture medium and nutrient availability. Under static cultivation conditions, the surface area-to-volume ratio significantly influences oxygen availability; a larger surface area favors oxygen consumption. Since AAB are aerobic microorganisms, oxygen supply is essential for cell growth. In this case, the cells are suggested to use the synthesized bacterial cellulose as a support to reach the air–liquid interface, which improves their access to oxygen. In contrast, aeration in the agitated method provides a greater oxygen supply and improves the availability of nutrients, thereby promoting cell growth (*34*, *103*, *154*, *155*).

Despite the higher oxygen diffusion under agitation, bacterial cellulose production can be negatively affected by the occurrence of mutant cells (non-producing cells), the formation of by-products and simultaneous production of water-soluble polysaccharides (WSPS), resulting in lower production than the static method (*38*, *103*, *153*). The formation of by-products is a consequence of the nutrient consumption, carbon source metabolization and aeration. For example, when using glucose as a carbon source, Chen *et al*. (*16*) reported higher glucose consumption and gluconic acid production in the shaking system. Krusong *et al*. (*156*) observed that gluconic acid production increased with the aeration rate, while bacterial cellulose production and cell content were reduced. In addition to these limiting-factors, recent research confirms the ability of some AAB strains to grow and produce bacterial cellulose under agitation, and the yield could be similar or higher than the static method (*24*, *71*, *103*).

## DOWNSTREAM METHODS OF ISOLATION AND PURIFICATION OF BACTERIAL CELLULOSE

The downstream processes for isolating and purifying bacterial cellulose can be divided into three main steps: harvesting, purification, and drying. At the end of the fermentation step, the produced bacterial cellulose is harvested from the liquid medium and separated by centrifugation or filtration (*32*, *71*, *153*).

The harvested bacterial cellulose must be purified to remove residues from the culture medium and cells, as these materials can affect the properties of the biopolymer, such as crystallinity and color, and can also lead to contamination of the bacterial cellulose (*157*, *158*). The conventional approach is alkaline treatment. In this method, bacterial cellulose is purified in an aqueous solution of sodium hydroxide and then neutralized by washing with distilled water. To achieve optimal purification efficiency, literature suggests various combinations of NaOH concentration, temperature and time. Typically, bacterial cellulose is purified with 0.1 to 1 M NaOH solution at 80 to 90 ºC, for 30 to 60 min (*10*, *17*, *100*). Alkaline treatment is suggested to be able to remove the remaining nutrients from the culture medium and to lyse bacterial cells attached to the bacterial cellulose (*102*).

## BIOPROCESS CONTROL, OPTIMIZATION AND SCALE-UP

As already mentioned, bacterial cellulose has unique properties for industrial applications. However, the implementation of a highly productive bioprocess is essential to scale up biopolymer production. Any fermentation process can be optimized by using statistical tools to evaluate the effect of critical parameters on the production or properties of bacterial cellulose. In addition to the one-factor-at-a-time approach, statistical optimization is often used to define the ideal conditions for bacterial cellulose production by analyzing a wide range of parameters, including the interaction of process variables.

Studies highlight the use of innovative substrates like enzymatically hydrolyzed prickly pear peels (PPP), which yield 6.01 g/L of bacterial cellulose under optimized conditions (68 % PPP substrate, pH=4, 20 °C, 11 days). Functionalized bacterial cellulose membranes loaded with fruit by-products, such as pomegranate peel extract, showed antimicrobial properties and extended the shelf life of strawberries, highlighting their potential for sustainable packaging (*159*).

Statistical models offer time- and cost-efficient alternatives for exploring fermentation conditions aimed at large-scale production. Considering their influence on bacterial cellulose yield, the most important parameters used in bioprocess optimization include the type and concentration of carbon and nitrogen sources, ethanol, pH, temperature, cultivation method, rotation speed, inoculum concentration and volume of culture medium (*12*, *32*, *141*, *160*).

Statistical optimization techniques, such as Plackett-Burman and Box-Behnken designs, have refined parameters like yeast extract concentration, temperature and incubation time, to significantly increase bacterial cellulose yield. For instance, *Gluconacetobacter hansenii* ATCC 23769 achieved 2.91 g/L bacterial cellulose under optimized conditions. A comprehensive characterization of bacterial cellulose membranes revealed high purity, crystallinity and thermal stability, which emphasizes their suitability for various applications, from packaging to biomedical and environmental uses (*161*).

Currently, the limitations of large-scale implementation are mainly related to raw material costs, energy and water consumption, formation of by-products, metabolism of carbon sources and reproducibility of yields obtained in the first stages (*105*, *144*). Scale-up studies and alternative modes of operation have been successfully carried out with different nutrient sources and acetic acid bacteria, confirming that this bioprocess can be further explored to improve the application of bacterial cellulose (*102*, *162*).

## CONCLUSIONS

Bacterial cellulose is a high-value product obtained by acetic acid fermentation and has the potential for wide use in industrial applications due to its unique properties. To achieve this, the key challenge is to optimize the yield to create a production system that can meet industrial demand. The development of a profitable bioprocess requires consideration of the interaction between the three key elements of fermentation: the strain, the culture medium and the cultivation method. This review aims to present the general aspects of bacterial cellulose production and summarize the main challenges and strategies to increase production and reduce bioprocess costs. The results presented in this review provide insights into alternatives to improve bacterial cellulose production. The development of a bioprocess for bacterial cellulose production requires: *i*) a highly productive strain, either wild-type or genetically engineered, *ii*) a low-cost nutrient source, achievable by using agro-industrial waste or substituting carbon sources in a synthetic medium and *iii*) an optimized cultivation method. An effective combination of these strategies needs to be explored to ensure bacterial cellulose production and its application on an industrial scale.
